# Editorial: Improving Working Memory in Learning and Intellectual Disabilities

**DOI:** 10.3389/fpsyg.2016.00725

**Published:** 2016-05-13

**Authors:** Silvia Lanfranchi, Barbara Carretti

**Affiliations:** ^1^Department of Developmental and Socialization Psychology, University of PadovaPadova, Italy; ^2^Department of General Psychology, University of PadovaPadova, Italy

**Keywords:** intellectual disabilities, learning disabilities, ADHD, working memory training, executive functions, transfer effects, maintenance effects, school outcomes

## Introduction

Working memory (WM) has been defined as a system for temporarily retaining and manipulating information while performing a variety of cognitive tasks (Baddeley, 1986). To date, the crucial role of WM in activities of everyday life (including reading, writing, arithmetic, learning, language-processing, orientation, imagination) has been demonstrated in an impressive body of research. Several studies have shown an impairment in WM in individuals with learning disabilities (LD) or intellectual disabilities (ID, e.g., Lanfranchi et al., 2004; LD, Peng and Fuchs, 2016).

Given its core role in cognition, the feasibility of training WM has emerged in the literature as a crucial issue, with efforts focusing on analyzing whether and how improving WM might affect cognitive processes associated with WM as well. The results have been contradictory so far, however, with some studies finding WM training effective in producing improvements in the trained task, but few reporting transfer effects to allied cognitive processes, and even fewer identifying any maintenance effects, when investigated (see Melby-Lervåg and Hulme, 2013, for example).

Starting from this literature, the aim of the research discussed here is to add new evidence on the direct and transfer effects of WM training in individuals with LD or ID. Several key points have emerged concerning WM training in these particular populations, as summarized in the following paragraphs.

## Efficacy of WM training: Specific or transfer effects?

The results of the studies presented in this research topic seem to indicate that WM is trainable in LD and ID, albeit with some differences coming to light depending on the type of training procedures used. All the research articles showed direct effects of the training considered on the WM task directly trained. However, few of these studies explored and demonstrated the stability of these gains over time (Pulina et al.; Orsolini) and only some of them identified transfer effects. The latter effects were only found for some variables (only for certain aspects of memory not directly trained, e.g., Orsolini; Ottersen and Grill; Pulina et al.), and not for all participants (e.g., Costa et al.), and they did not always persist over time (e.g., Orsolini). Similar results emerged from the meta-analysis conducted by Danielsson et al. on the effects of WM training on individuals with ID.

## How are WM processes trained?

No consensus has been reached as yet on how best to train WM. There is a certain variability in the WM training procedures adopted to date: some studies have proposed activities focusing on a specific domain (as in the case of Pulina et al.); others have taken a multi-domain approach (e.g., Holmes et al.); others again have suggested that the best solution is to combine the two, i.e., practice with verbal and visuospatial WM together with learning new strategies to use in WM tasks (Danielsson et al.). Another interesting approach, proposed here by several authors (Swanson; Garcia-Madruga et al.), is to combine WM exercises within the context of the learning skill needing to be improved (in the cited articles, this was done to improve problem solving and reading comprehension); the idea is to enhance the likelihood of training gains being transferred to other abilities not trained directly.

Another interesting issue regards the attempt to bring WM training to school, providing the training during regular classroom activities (e.g., Traverso et al.; Re et al.; Costa et al.), or asking teachers to monitor and stimulate children to practice the strategies learned during the WM training (as in van der Donk). This is an important aspect because most WM trainings involve individual sessions separately from the normal school activities. But any training needs to be repeated regularly over a certain period of time in order to be effective, and this could prove an organizational problem for the families of children with LD or ID. Practical obstacles could make parents unwilling or unable to ensure that their children attend training programs. The experiences reported in the present research topic testify to the feasibility of organizing activities that focus on WM and executive processes in the context of normal school activities. This is an aspect that appears to be particularly relevant also in terms of the potential effects on academic outcomes.

In the same vein, the study by Pulina et al. examined the feasibility of parents training their children's WM directly, under the supervision of an expert. The results of this first study are encouraging, suggesting that this might be a good way to train children in a more ecological setting. Of course, more evidence is needed in this sense to confirm as much.

Analyzing the literature on WM in children with LD and ID gives the impression that, depending on the etiology of a given deficit, there might be a particular profile of WM impairment, and children might consequently benefit from different training programs that place more emphasis on some aspects rather than on others. Several studies in this research topic indicate that training programs should be adapted to the type of children with which they are used. For example, Ottersen and Grill showed that a group of children with ID benefited more from a cognitive training that lasted longer and involved less demanding tasks than those applied to children without ID. Pulina et al. also demonstrated the efficacy of a training program in which the material was adapted to the cognitive profile of individuals with Down syndrome.

## Who benefits from training?

The findings of the studies reported in this research topic suggest that any training-induced improvement in WM is not homogeneous for all individuals. It seems to depend on several factors relating to the type of training and to certain individual characteristics.

Concerning the type of training, Titz and Karbach (2014) recently suggested that strategic training produced magnification effects (thereby augmenting individual differences), in the memory domain at least, whereas process-based training (focusing on WM and executive functions, for example) promoted compensation effects (thus reducing individual differences, and consequently benefiting lower-performing individuals). The results of the studies described in this research topic are consistent with this view. In the study by Costa et al., for example, a school-based treatment targeting visuo-spatial WM was administered to two individuals with DS for 6 weeks, after which one of them showed good direct and transfer effects, the other only weak direct effects. The two apparently had different baseline WM levels, and the one with a worse WM at the start achieved greater improvements. These findings suggest that training activities could be particularly effective in children with an initially worse performance, which is in line with a compensation effect (see also Holmes et al.).

In contrast, Swanson showed that children with math disability took more or less advantage of a different strategic training depending on their initial level of WM: children performing at a higher level initially improved to a greater extent after the training. In this case, Swanson's results point to an amplification effect of strategic training. Interestingly, Holmes et al. reported larger transfer effects in children with higher baseline IQ levels.

As concerns individual factors, Alesi et al. explored the role of motivational beliefs and showed that a verbal WM training was more effective for a child with an incremental theory of intelligence than for a child with a static representation of intelligence.

Consistently with these results, Morra and Borella suggests that future studies on the efficacy of WM training should consider baseline performance in WM tasks (and possibly other cognitive and motivational variables too) as an indication of an individual's chances of benefiting from training. For instance, it may be that a minimal WM capacity is needed for any training to generate an improvement, or that there is an ideal capacity level (neither too high nor too low) that makes the training likely to work better.

Considering all these aspects, it appears particularly relevant the suggestion advanced by Konen and Karbach to study the intra-individual dynamics of cognitive training data in order to better elucidate which variables make a given type of training the most effective for a given individual.

## Conclusion

In the light of all the aspects emerging from the papers reported in this research topic, we are convinced that more research is needed to establish how WM can be trained effectively in individuals with ID and LD.

We hope that all the points raised here might be helpful to all those researchers planning to approach the field of WM training in individuals with LD and ID in the future.

## Author contributions

All authors listed, have made substantial, direct and intellectual contribution to the work, and approved it for publication.

## Funding

This work was supported by a grant awarded by the University of Padova to SL (CPDA127939).

### Conflict of interest statement

The authors declare that the research was conducted in the absence of any commercial or financial relationships that could be construed as a potential conflict of interest.
